# Supplementary material for: Epigenomic charting and functional annotation of risk loci in renal cell carcinoma
Source: Nat Commun. 2023 Jan 21;14:346. doi: 10.1038/s41467-023-35833-5 (PMC9867739; doi:10.1038/s41467-023-35833-5)
Supplement: Supplementary file 1 — Supplementary Information [file 41467_2023_35833_MOESM1_ESM.pdf]

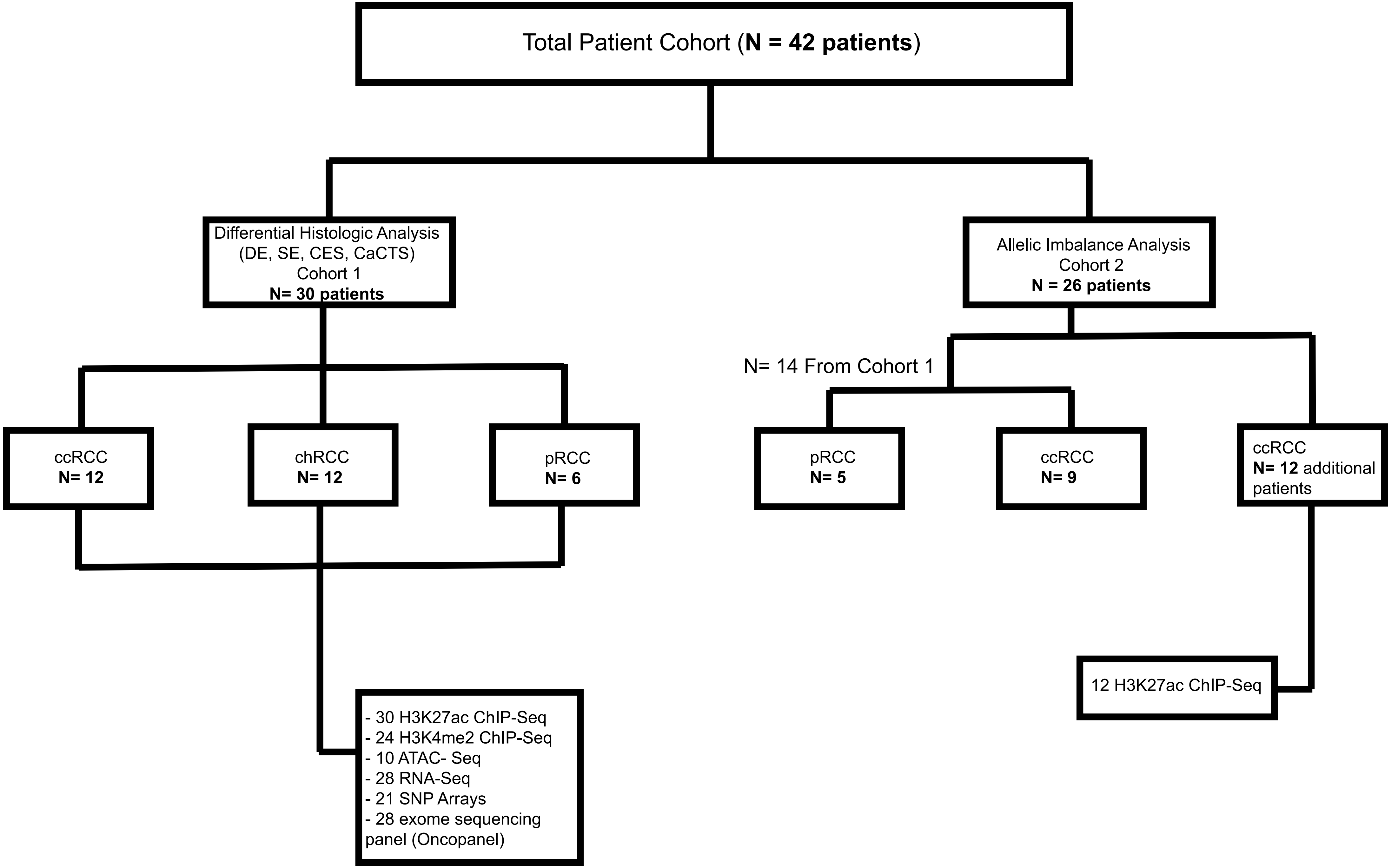

Supplementary Figure S1. CONSORT Diagram. N: number. RCC: renal cell carcinoma. ccRCC: clear cell RCC. chRCC: chromophobe RCC. pRCC: papillary RCC. DE: Differential Expression. SE: Super Enhancer. CES: Clique Enrichment Score. CaCTS: Cancer Core Transcription factor Specificity. ChIP-seq: Chromatin Immunoprecipitation Sequnecing. ATAC-seq: Assay for Transposase-Accessible Chromatin Sequencing.

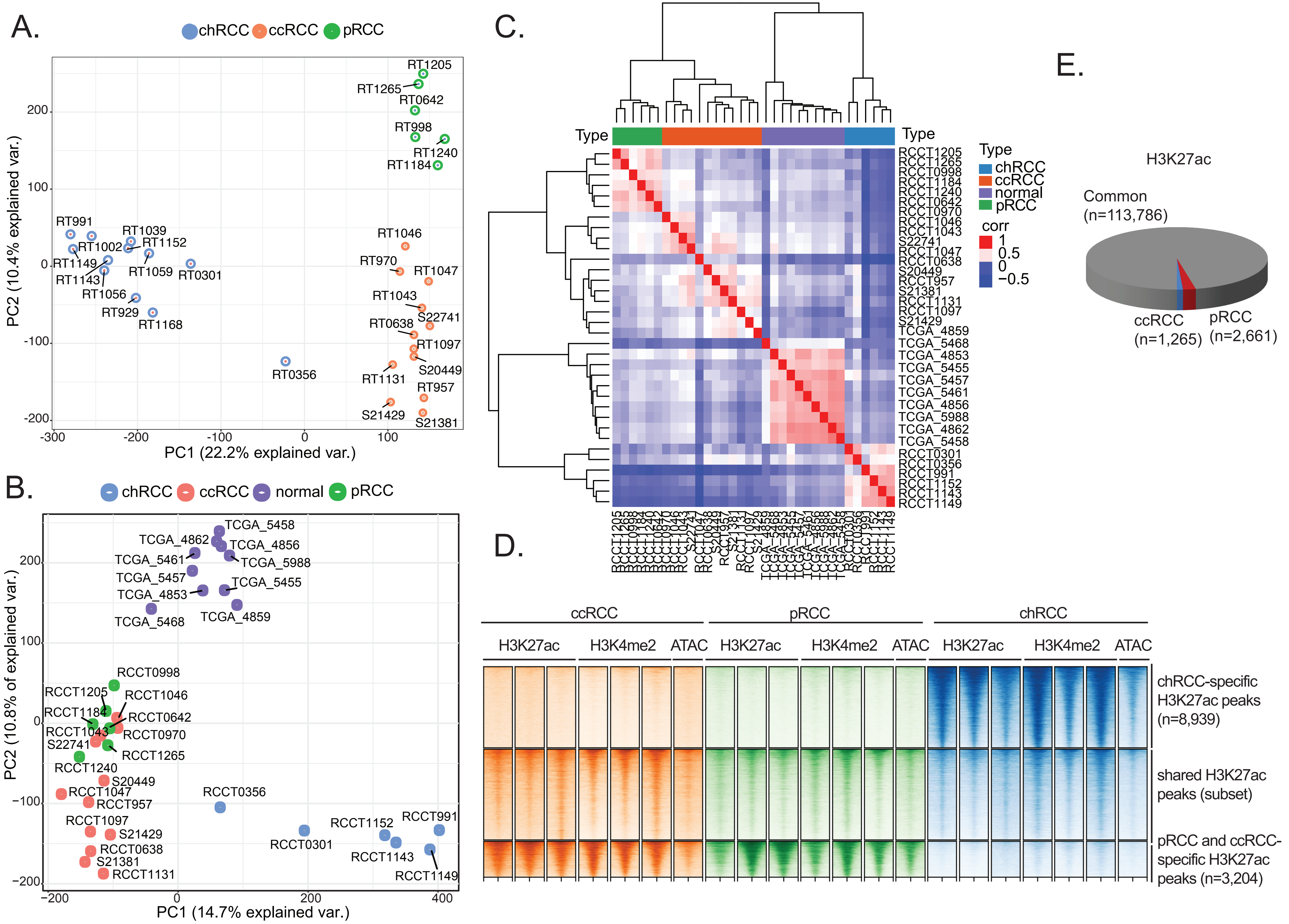

Supplementary Figure S2. Distribution of H3K4me2, H3K27Ac and ATAC-Seq epigenetic marks across RCC histologies. A. Principal component analysis of DFCI ccRCC, chRCC, and pRCC based on H3K27ac profiles. B. Principal component analysis of DFCI ccRCC, chRCC, pRCC, and TCGA normal kidney tissue based on H3K27ac profiles. C. Unsupervised hierarchical clustering of DFCI ccRCC, chRCC, pRCC, and TCGA normal kidney tissue based on H3K27ac peaks. Each column is a different sample. D. Heatmaps of normalized H3K27ac, H3K4me2, and ATAC tag densities at differentially H3K27-acetylated regions (±2kb from peak center) between chRCC, pRCC, and ccRCC. Each column is a different sample. Few representative samples from each histology are shown. E. Pie chart of common and histology-specific H3K27ac peaks between ccRCC and pRCC. ChIP-seq: Chromatin Immunoprecipitation Sequnecing. ATAC-seq: Assay for Transposase-Accessible Chromatin Sequencing. RCC: renal cell carcinoma. ccRCC: clear cell RCC. chRCC: chromophobe RCC. pRCC: papillary RCC. PC: Principal Component.

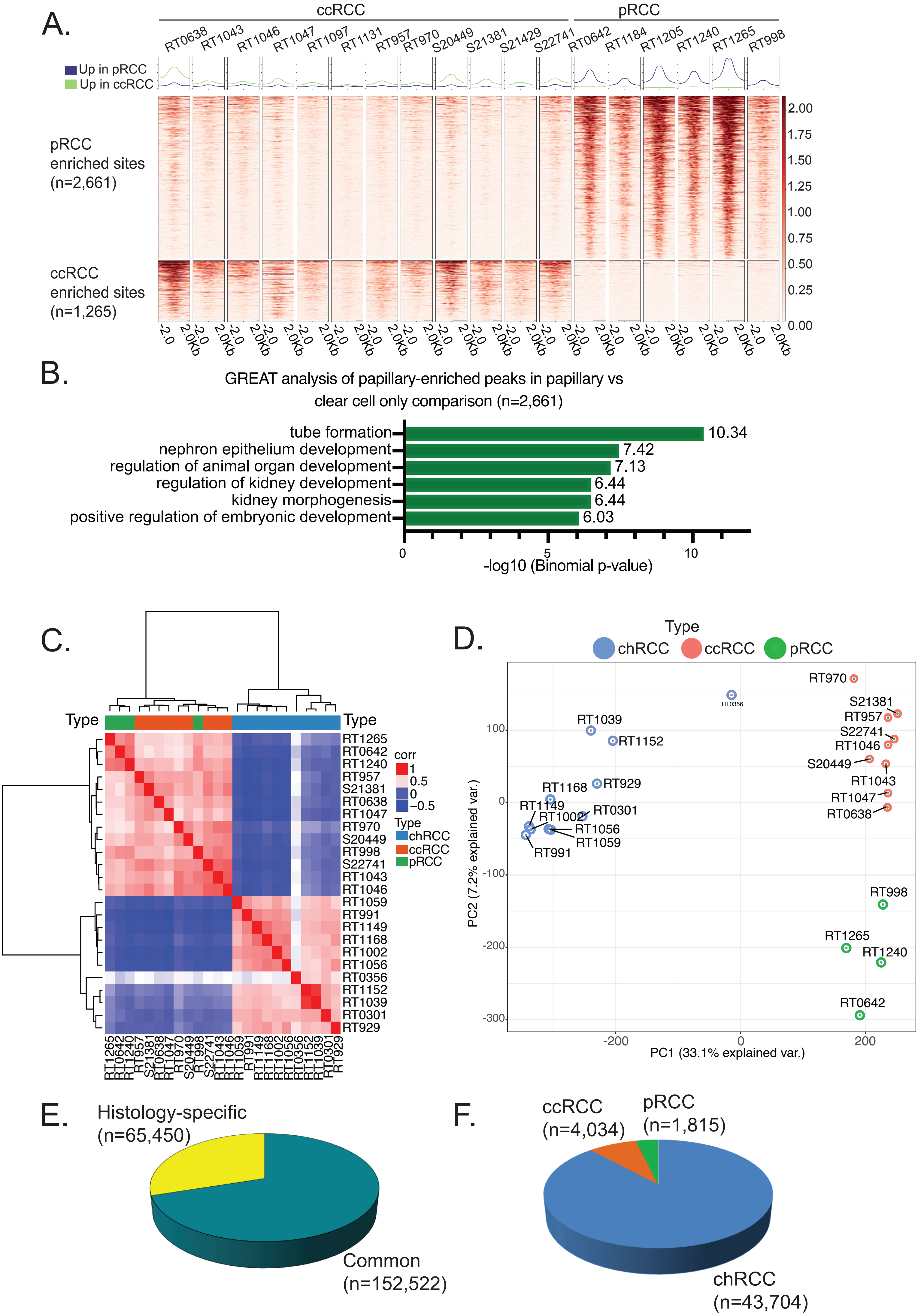

Supplementary Figure S3. Differential H3K27Ac and H3K4me2 peaks across different RCC histologies. A. Heatmaps of normalized H3K27ac tag densities at differentially H3K27-acetylated regions (±2kb from peak center) between pRCC and ccRCC. B. GREAT analysis of papillary-enriched peaks in papillary vs clear cell only comparison (n=2,661). GREAT calculates statistical enrichments for association between genomic regions and annotations. Two-sided p-values are shown. C. Hierarchical clustering of chRCC, ccRCC, and pRCC based on sample-to-sample pairwise correlation of the H3K4me2 ChIP-seq signals (red – strong correlation, pink – intermediate correlation, blue– opposite correlation). The diagonal of cells with red outlines denotes the line of identity. D. Principal component analysis of ccRCC, chRCC, and pRCC based on H3K4me2 profiles. E. Pie chart of numbers of H3K4me2 peaks among n= 24 samples (n= 11 chRCC; n= 9 ccRCC; n= 4 pRCC). F. Pie chart of numbers of histology-specific H3K4me2 peaks among n= 24 samples (n= 11 chRCC; n= 9 ccRCC; n= 4 pRCC). RCC: renal cell carcinoma. ccRCC: clear cell RCC. chRCC: chromophobe RCC. pRCC: papillary RCC. PC: Principal Component.

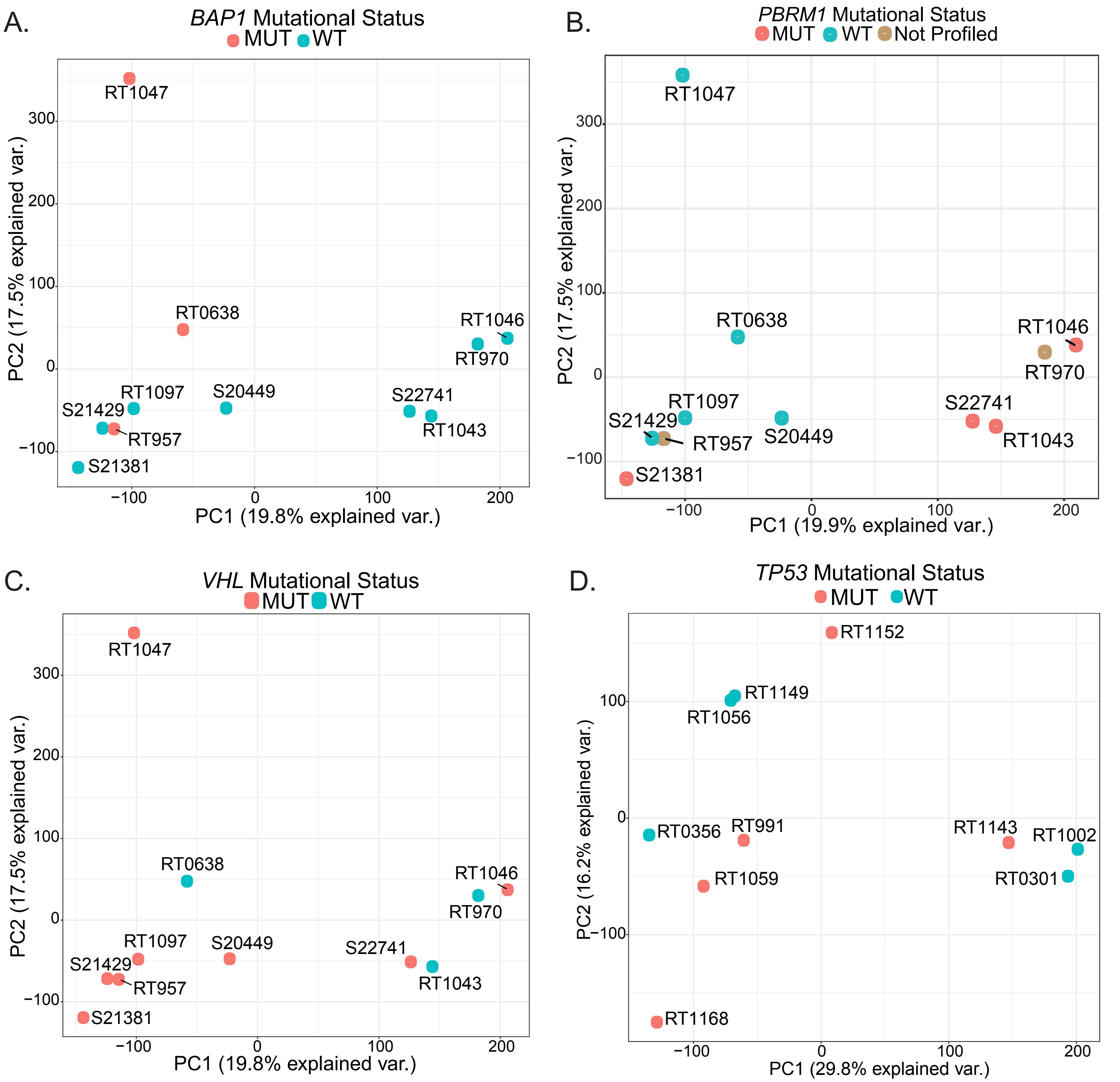

Supplementary Figure S4. Principal component analysis of H3K27ac ChIP-Seq data of ccRCC and chRCC samples based on mutational status. ABC. Mutational status of BAP1, PBRM1, and VHL genes in ccRCC samples. D. Mutational status of TP53 gene in chRCC samples. MUT: mutant; WT: Wild Type. PC: Principal component.

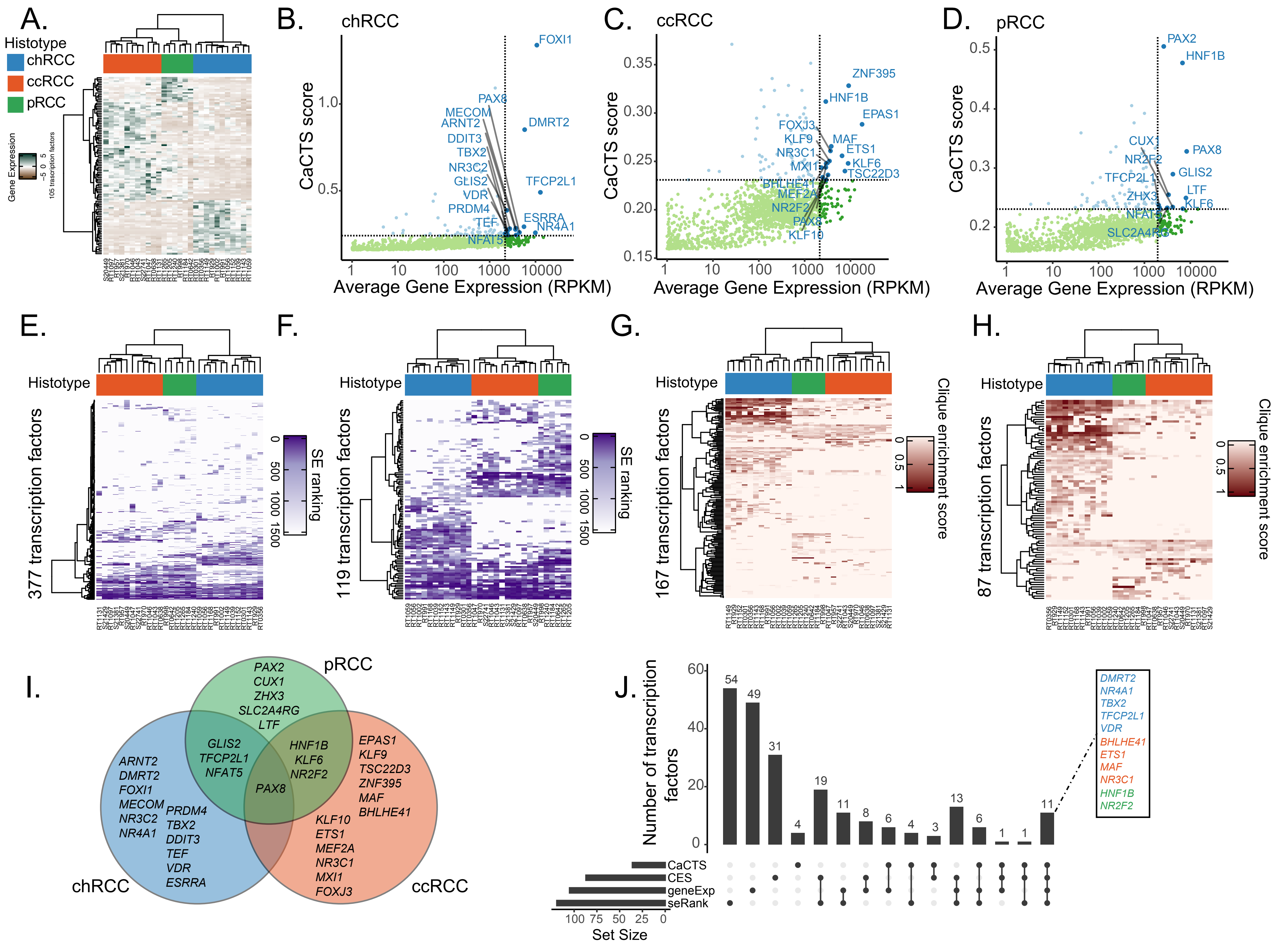

Supplementary Figure S5. TF Expression analysis among RCC histologies. A. Heatmap depicting sample-to-sample clustering based on differentially expressed TFs (n=105) across 28 RCC samples, FDR=10%, Minimum TPM =10, Minimum fold change=2. BCD. CaCTS score analysis for chRCC, ccRCC, and pRCC, respectively. E. Heatmap depicting SE-associated TFs (n=377 genes) across 30 RCC samples. Gene-sample pairs without an SE rank score were assigned a value of 1500. F. Heatmap depicting TFs with differentially active SE-associated TFs (n=119 genes) across 30 RCC samples. FDR=10%, Max SE rank is 1500. G. Heatmap depicting sample clustering based on Clique Enrichment Scores (CES) of all TFs (n=167) with a CES > 0 in at least one sample. H. Heatmap depicting sample clustering based on TFs with differential CES by histology (n=87). ANOVA test. I. Venn diagram showing common and histology-specific master TFs using the CaCTS algorithm. J. UpsetR plot showing the number of transcription factors identified using each individual method (CaCTS, differential CES, differential gene expression, and differential SE rank). RCC: renal cell carcinoma. ccRCC: clear cell RCC. chRCC: chromophobe RCC. pRCC: papillary RCC. SE: Super enhancer. CaCTS: Cancer Core Transcription Factor Specificity. geneExp: Gene expression. TF: Transcription Factor. Source data are provided as a Source Data file.

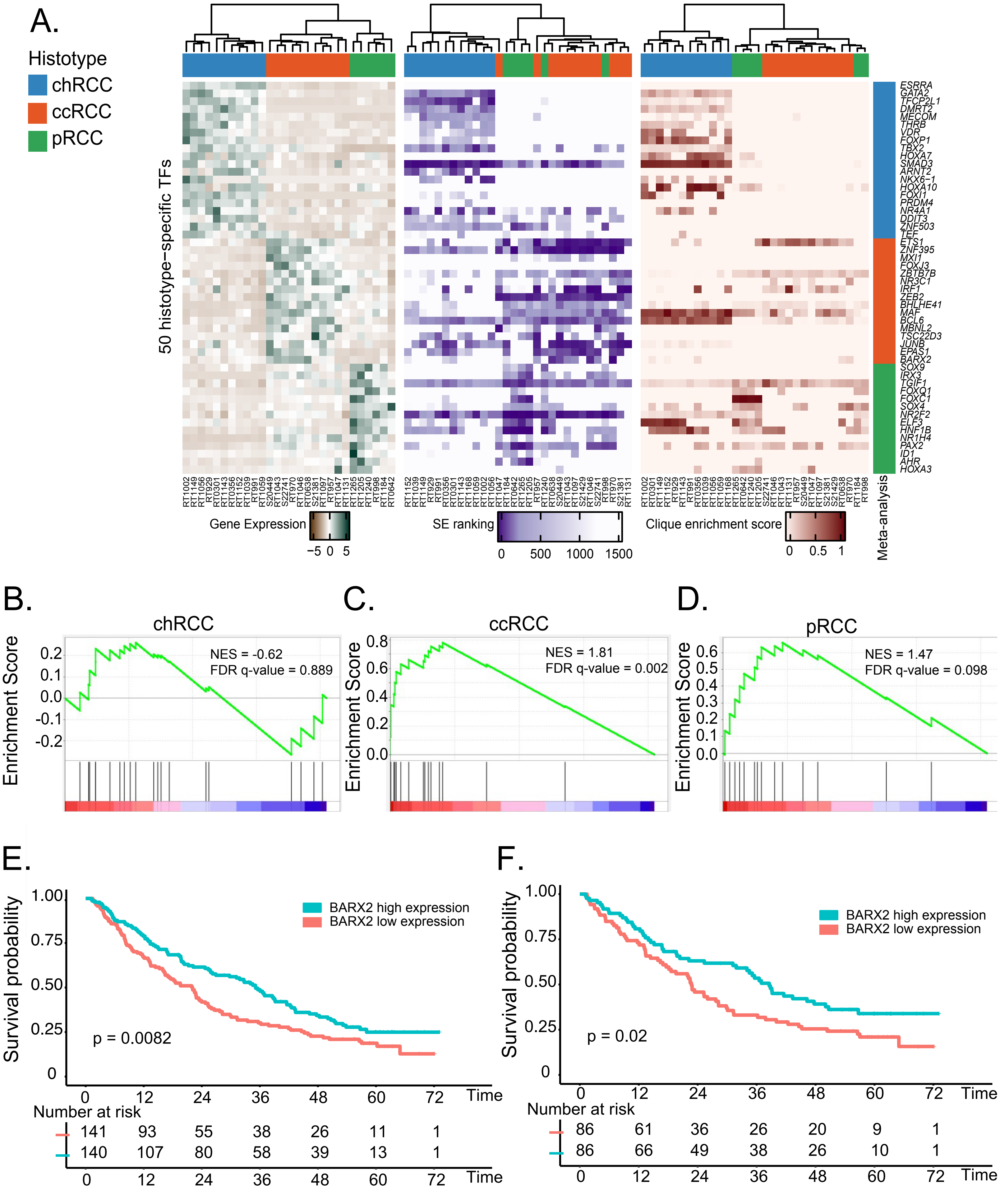

Supplementary Figure S6. Tumor-specificity of master TFs and clinical associations. A. Sample-to-sample clustering by the gene expression, SE rank and CES of the 50 candidate master TFs. GE: Gene Expression. BCD. Gene set enrichment analysis (GSEA) of 50 master TFs in the tumor tissue compared to the normal counterpart in TCGA samples. EF. Overall survival associations according to BARX2 median expression in CheckMate (009/010/025) ccRCC. Panel E includes patients treated with either nivolumab or everolimus (n=281). Panel F includes patients treated with nivolumab only (n=172). For E and F, two-sided unadjusted P values are of the Wald χ2 test from the Cox regression analysis. pRCC: papillary RCC. SE: Super enhancer.

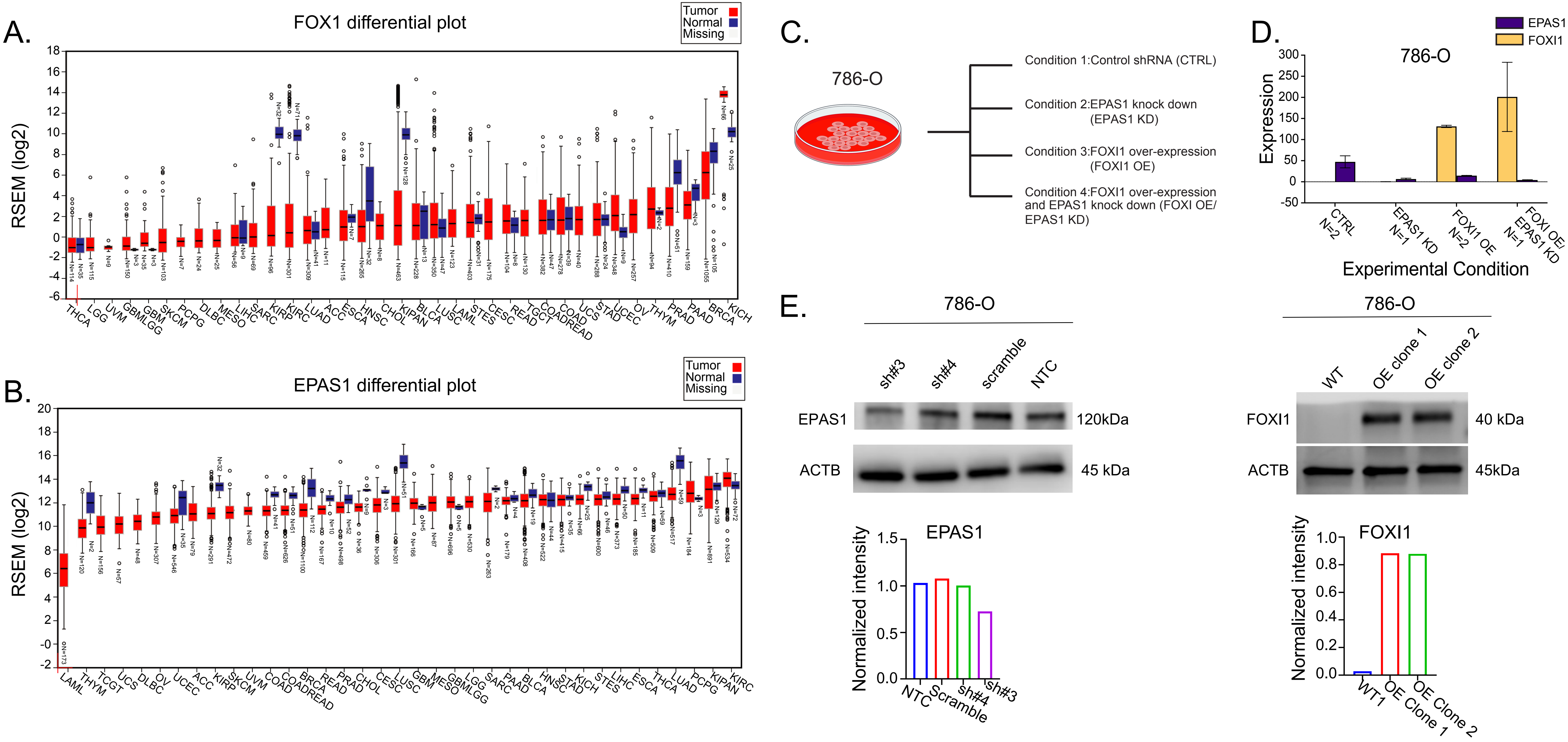

Supplementary Figure S7 Experimental design for candidate master transcription factor perturbations in 786-O. AB. Pancancer expression of FOXI1 and EPAS1, respectively. Source: The Cancer Genome Atlas. http://firebrowse.org/viewGene.html?gene=foxi1. The number of independent biological samples is indicated next to each box plot. Box boundaries correspond to 1st and 3rd quartiles; whiskers extend to a maximum of 1.5x the inter-quartile range. C. Experimental Setup. D. Expression levels of EPAS1 and FOXI1 in 786-O across the different experimental conditions. The number of independent biological samples is indicated next to each experimental group. Box boundaries correspond to 1st and 3rd quartiles; whiskers extend to a maximum of 1.5x the inter-quartile range. E. Western blot, with quantification, to confirm knockdown of EPAS1 (Left) and overexpression of FOXI1 (right) in 786-O cells. Densitometry for EPAS1 and FOXI1 was performed using ImageJ and normalized to ACTB. Two technical replicates were performed for each condition. Numbers next to western blots indicate molecular weight markers (kD). LAML: Acute Myeloid Leukemia. ACC: Adrenocortical carcinoma. BLCA: LGG: Brain Lower Grade Glioma. BRCA: Breast invasive carcinoma. CESC: Cervical squamous cell carcinoma and endocervical adenocarcinoma. CHOL: Cholangiocarcinoma. LCML: Chronic Myelogenous Leukemia. COAD: Colon adenocarcinoma. ESCA: Esophageal carcinoma. GBM: Glioblastoma multiforme. HNSC: Head and Neck squamous cell carcinoma. KICH: Kidney Chromophobe. KIRC: Kidney renal clear cell carcinoma. KIRP: Kidney renal papillary cell carcinoma. LIHC: Liver hepatocellular carcinoma. LUAD: Lung adenocarcinoma. LUSC: Lung squamous cell carcinoma. DLBC: Lymphoid Neoplasm Diffuse Large B-cell Lymphoma. MESO: Mesothelioma. OV: Ovarian serous cystadenocarcinoma. PAAD: Pancreatic adenocarcinoma. PCPG: Pheochromocytoma and Paraganglioma. PRAD: Prostate adenocarcinoma. READ: Rectum adenocarcinoma. SARC: Sarcoma. SKCM: Skin Cutaneous Melanoma. STAD: Stomach adenocarcinoma. TGCT: Testicular Germ Cell Tumors. THYM: Thymoma. UCS: Uterine Carcinosarcoma. UCEC: Uterine Corpus Endometrial Carcinoma. UVM: Uveal Melanoma. KIPAN: Pan-kidney cohort (KICH+KIRC+KIRP). sh: short hairpin. NTC: non-targeting control. WT: Wild type. OE: overexpression. Source data are provided as a Source Data file.

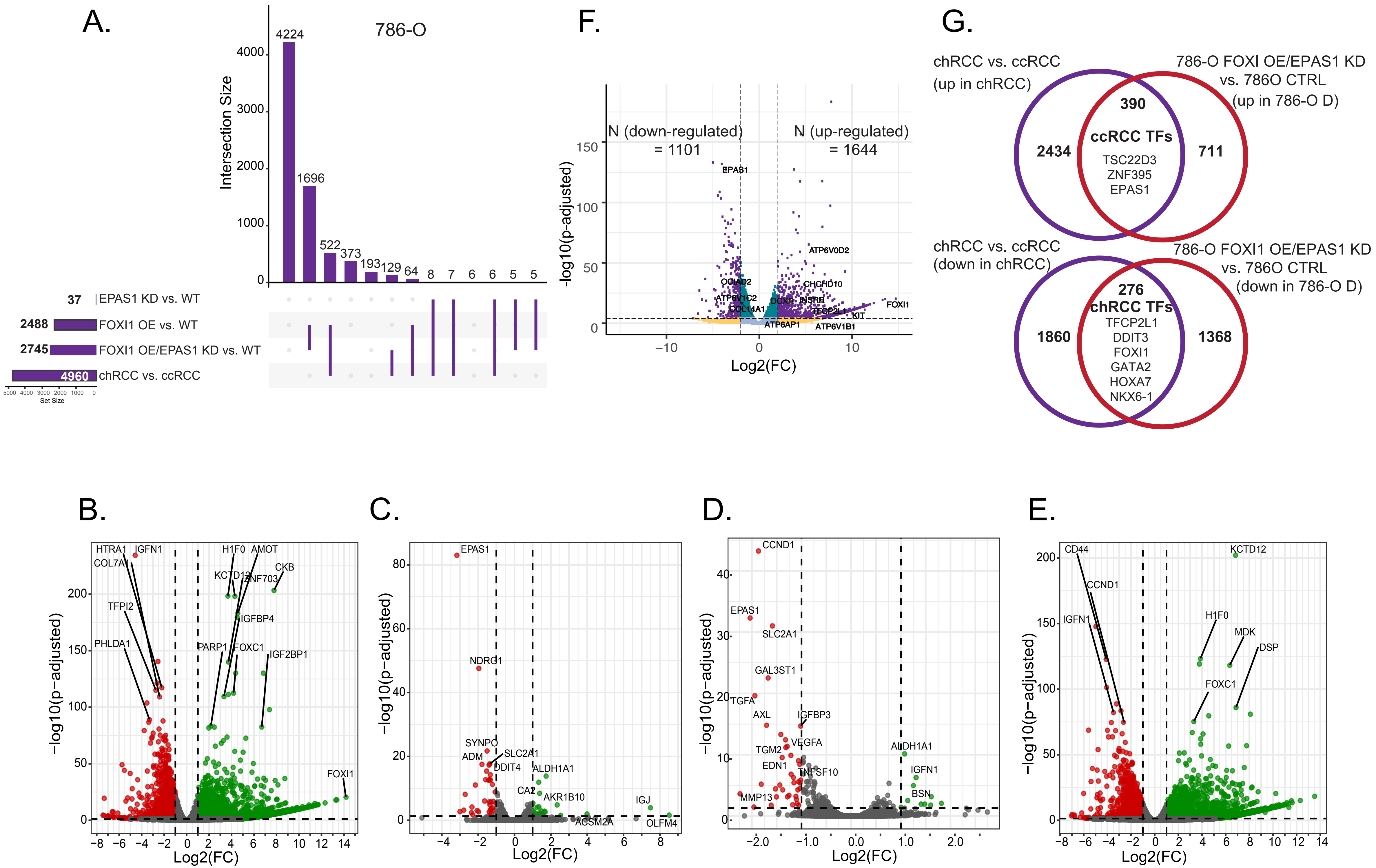

Supplementary Figure S8. Transcriptional changes across different experimental conditions in 786-O. A. UpsetR plot showing the number of differentially expressed genes under each experimental condition for cell line 786-O. B. Volcano plot of RNA-Seq data showing differentially expressed genes plotted according to log 2 fold-change and -log10(p value) for 786-O CTRL vs. FOXI1 OE. C. Volcano plot of RNA-Seq data showing differentially expressed genes plotted according to log 2 fold-change and -log10(p value) for 786-O CTRL vs. 786-O EPAS1 KD. D. Volcano plot of RNA-Seq data showing differentially expressed genes plotted according to log 2 fold-change and -log10(p value) for 786-O OE vs. 786-O FOXI1 OE/EPAS1 KD. E. Volcano plot of RNA-Seq data showing differentially expressed genes plotted according to log 2 fold-change and -log10(p value) for 786-O EPAS1 KD vs. 786-O FOXI1 OE/EPAS1 KD. Genes over/under expressed are shown in purple (fold-change > 1, FDR-corrected p-value < 0.001). A few genes of interest are highlighted. F. Volcano plot of RNA-Seq data showing differentially expressed genes plotted according to log 2 fold-change and -log10 (p value) for 786-O CTRL vs. 786-O FOXI1 OE/EPAS1 KD. For B-F, two-sided p-values were used and corrected for multiple comparison testing (FDR-adjusted p-value < 0.05). G. Venn diagram comparing differentially expressed genes between “chRCC vs. ccRCC” and “ 786-O CTRL vs. 786-O FOXI1 OE/EPAS1 KD”. Top Venn diagram refers to genes upregulated in chRCC vs. ccRCC. Bottom Venn diagram refers to genes downregulated in chRCC vs. ccRCC. Data shown for two experimental replicates. RCC: renal cell carcinoma. chRCC: chromophobe RCC. ccRCC: clear cell RCC. pRCC: papillary RCC. KD: Knock down. OE: Over expression. CTRL: control. FC: Fold Change.

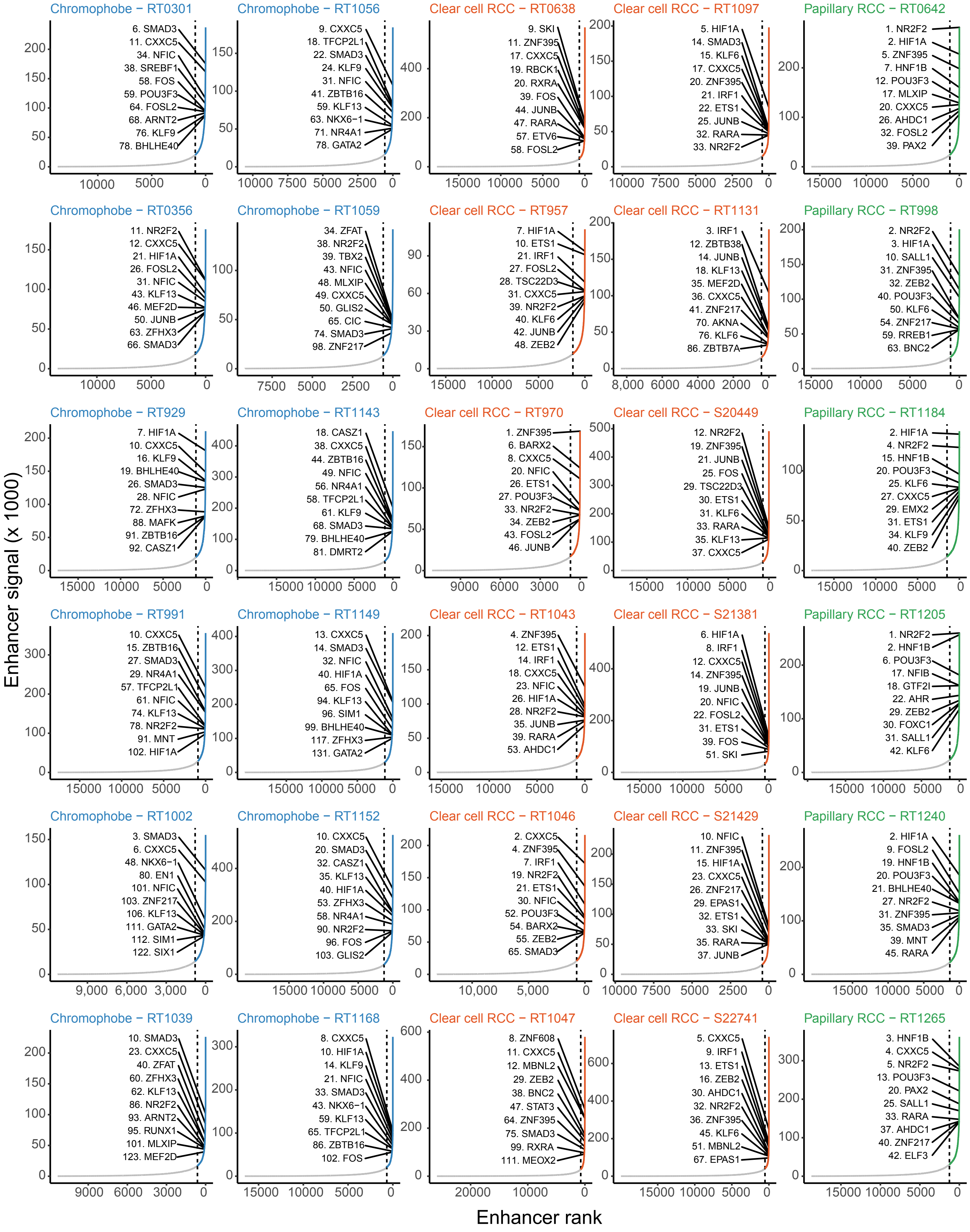

Supplementary Figure S9. Rank-ordered plots of all enhancer regions identified by H3K27ac ChIP-Seq and their ROSE scores. SE-associated TFs are shown for each (n=30) RCC sample. SE: Super enhancer. ChIP-Seq: Chromatin Immunoprecipitation Sequencing.
