# Supplementary material for: Epigenomic charting and functional annotation of risk loci in renal cell carcinoma
Source: Nat Commun. 2023 Jan 21;14:346. doi: 10.1038/s41467-023-35833-5 (PMC9867739; doi:10.1038/s41467-023-35833-5)
Supplement: Supplementary file 3 — Description of Additional Supplementary Files [file 41467_2023_35833_MOESM3_ESM.pdf]

Description of Additional Supplementary Files

Supplementary Data 1: Master table. Samples including clinical and pathological data, analyses performed, and mutation data.

Supplementary Data 2: Omics Platform. Summary of the assays performed on each sample.

Supplementary Data 3: SNV_Calls. SNV and small indel variants across 28 samples with Oncopanel sequencing data.

Supplementary Data 4: CNV_Calls. Copy number variant calls across 28 samples with Oncopanel sequencing data.

Supplementary Data 5: Gene expression. Normalized gene expression of RCC samples.

Supplementary Data 6: Differential Gene Expression. Log2 fold changes and adjusted p-values for differential gene expression pairwise comparisons across histologies (chromophobe RCC, clear cell RCC, papillary RCC).

Supplementary Data 7: CaCTS scores. List of CaCTS scores/ranks coupled with gene expression means/ranks for the CaCTS candidates in each RCC histological subtype.

Supplementary Data 8: Super-enhancer ranks. Super-enhancer rank of SE-associated genes for 30 samples. Each column represents one individual sample. Each row represents an individual gene. NA: Not applicable.

Supplementary Data 9: Differential SE ranks. Log2 fold changes and adjusted two-sided p-values for differential SE ranks pairwise comparisons across histologies (chRCC, ccRCC, pRCC).

Supplementary Data 10: CES. Full list of transcription factors with their corresponding clique enrichment score (CES) in RCC samples.

Supplementary Data 11: Differential CES. Log2 fold changes and adjusted p-values for differential CES pairwise comparisons across histologies (chRCC, ccRCC, pRCC).

Supplementary Data 12: Meta-analysis. Summary tables incorporating transcription factors identified in the meta-analysis approach (CES, SE rank, differential expression, and CaCTS). For each analysis (CES, SE rank, differential expression, and CaCTS), adjusted two-sided p-values are reported.

Supplementary Data 13: Survival Data from CheckMate trials. For all genes analyzed, two-sided unadjusted P-values and adjusted P-values (denoted as q-values) are of the Wald $\chi 2$ test from the Cox regression analysis.

Supplementary Data 14: RNA-Seq_Cell lines. Normalized gene expression data in HEK293 and 786-O cell lines. Gene expression data from replicates are shown for each of the four conditions.

Supplementary Data 15: 786O-FOXI1oevsWT.deseq - Differential gene expression between 786-O CTRL and 786-O FOXI1 OE. Log2 fold changes and adjusted two-sided p-values for differential SE ranks pairwise comparisons across conditions (786O-FOXI1OE vs 786-O CTRL).

Supplementary Data 16: 786O-EPAS1-KDvsWT.deseq - Differential gene expression between 786-O CTRL and 786-O EPAS1 KD. Log2 fold changes and adjusted two-sided p-values for differential SE ranks pairwise comparisons across conditions (786O-EPAS1 KD vs 786-O CTRL).

Supplementary Data 17: 786O-DvsFOXI1oe.deseq - Differential gene expression between 786-O FOXI1 OE and 786-O FOXI1 OE/EPAS1 KD. Log2 fold changes and adjusted two-sided p-values for differential SE ranks pairwise comparisons across conditions (786-O FOXI1 OE vs 786-O FOXI1 OE/EPAS1 KD).

Supplementary Data 18: 786O-DvsEPAS1kd.deseq - Differential gene expression between 786-O EPAS1 KD and 786-O FOXI1 OE/EPAS1 KD. Log2 fold changes and adjusted two-sided p-values for differential SE ranks pairwise comparisons across conditions (786-O EPAS1 KD vs 786-O FOXI1 OE/EPAS1 KD).

Supplementary Data 19: Cell Line & Tissue RNA. A. Comparative Analysis between differentially expressed genes between “chRCC vs. ccRCC” and “786-O CTRL vs. 786-O FOXI1 OE/EPAS1 KD”. B. Comparative Analysis between differentially expressed genes between “pRCC vs. ccRCC” and “786-O CTRL vs. 786-O FOXI1 OE/EPAS1 KD”. Unadjusted two-sided P-value calculated using Fisher’s Exact test. WT: Wild Type. D: Double cell line 786-O FOXI1 OE/EPAS1 KD

Supplementary Data 20: Allelically imbalanced H37K27ac peaks. List of all allelicaly imbalanced H3K27ac peaks with their respective chromosomal locations. Two-sided beta binomial p-values are shown.

Supplementary Data 21: List of 30 GWAS risk SNPs

Supplementary Data 22: Allele-specific expression of genes in the TCGA KIRC cohort.

Supplementary Data 23: SNP loci with chromatin allelic imbalance in the ccRCC DFCI cohort and the status of allele-specific expression of genes within 50Kb of respective SNP loci in TCGA KIRC cohort. Adjusted two-sided P-values are shown for chromatin allelic imbalance. Adjusted two-sided P-values<0.01 are significant for allele-specific expression.

Supplementary Data 24: Chromatin allelically balanced SNP loci in the ccRCC DFCI cohort and the status of allele-specific expression of genes within 50Kb of respective SNP loci in TCGA KIRC cohort. Adjusted two-sided P-values<0.01 are significant for allele-specific expression.

Supplementary Data 25: Enrichment analysis of allele-specific expression among chromatin allelically imbalanced versus balanced SNPs lying within the H3K27ac consensus peak set

Supplementary Data 26: ChIP-seq & ATAC-seq quality control measures

Supplementary Data 27: Oligonucleotide sequences for design of FOXI1 OE and EPAS1 shRNA knockout.
