# Supplementary material for: Epigenomic charting and functional annotation of risk loci in renal cell carcinoma
Source: Nat Commun. 2023 Jan 21;14:346. doi: 10.1038/s41467-023-35833-5 (PMC9867739; doi:10.1038/s41467-023-35833-5)
Supplement: Supplementary file 31 — Reporting Summary [file 41467_2023_35833_MOESM31_ESM.pdf]

nature portfolio

Corresponding author(s): Matthew Freedman

Last updated by author(s): 12/4/2022

Reporting Summary

Nature Portfolio wishes to improve the reproducibility of the work that we publish. This form provides structure for consistency and transparency in reporting. For further information on Nature Portfolio policies, see our Editorial Policies and the Editorial Policy Checklist.

Statistics

For all statistical analyses, confirm that the following items are present in the figure legend, table legend, main text, or Methods section.

n/a	Confirmed	
☐	☒	The exact sample size (*n*) for each experimental group/condition, given as a discrete number and unit of measurement
☐	☒	A statement on whether measurements were taken from distinct samples or whether the same sample was measured repeatedly
☐	☒	The statistical test(s) used AND whether they are one- or two-sided<br>*Only common tests should be described solely by name; describe more complex techniques in the Methods section.*
☐	☒	A description of all covariates tested
☐	☒	A description of any assumptions or corrections, such as tests of normality and adjustment for multiple comparisons
☐	☒	A full description of the statistical parameters including central tendency (e.g. means) or other basic estimates (e.g. regression coefficient) AND variation (e.g. standard deviation) or associated estimates of uncertainty (e.g. confidence intervals)
☐	☒	For null hypothesis testing, the test statistic (e.g. *F*, *t*, *r*) with confidence intervals, effect sizes, degrees of freedom and *P* value noted<br>*Give P values as exact values whenever suitable.*
☒	☐	For Bayesian analysis, information on the choice of priors and Markov chain Monte Carlo settings
☒	☐	For hierarchical and complex designs, identification of the appropriate level for tests and full reporting of outcomes
☐	☒	Estimates of effect sizes (e.g. Cohen's *d*, Pearson's *r*), indicating how they were calculated

Our web collection on statistics for biologists contains articles on many of the points above.

Software and code

Policy information about availability of computer code

Data collection	Data collection and analyses were performed R version 4.0.1. Raw Illumina output was converted to fastq format using Illumina bcl2fastq v2.18
Data analysis	Data collection and analyses were performed R version 4.0.1. All software or code used in the study, along with version numbers, is described in the methods section. We used: 1) HOMER version 4.7; 2) the Burrows-Wheeler Aligner (BWA) version 0.7.15; 3) DEseq 1.14.1; 4) GREAT v3.0; 5) MACS v2.1.1.20140616; 6) prcomp R function; 7) BEDTools v2.26.0.; 8) R package jsd version 0.1; 8) ROSE2; 9) Genome Analysis Toolkit v3.8

For manuscripts utilizing custom algorithms or software that are central to the research but not yet described in published literature, software must be made available to editors and reviewers. We strongly encourage code deposition in a community repository (e.g. GitHub). See the Nature Portfolio guidelines for submitting code & software for further information.

Data

Policy information about availability of data

All manuscripts must include a data availability statement. This statement should provide the following information, where applicable:

- Accession codes, unique identifiers, or web links for publicly available datasets
- A description of any restrictions on data availability
- For clinical datasets or third party data, please ensure that the statement adheres to our policy

Sequencing reads are aligned to the human genome build hg19. The DFCI datasets generated in this study have been deposited in the Gene Expression Omnibus

(GEO) database under accession code (GSE188486; https://www.ncbi.nlm.nih.gov/geo/query/acc.cgi?acc=GSE188486). The TCGA Kidney Renal Clear Cell Carcinoma (KIRC) publicly available data used in this study are available in a public repository from the Broad Institute Firehose Pipeline (http://gdac.broadinstitute.org). All clinical and correlative sequencing data from the publicly available CheckMate 009/010/025 clinical trials are made separately available as part of the accompanying paper (European Genome-Phenome Archive (https://ega-archive.org/; accession numbers EGAS00001004291 and EGAS00001004292)121. The remaining data are available within the Article, Supplementary Information or Source Data file. Any other queries about the data used in this study should be directed to the corresponding authors of this study.

Human research participants

Policy information about studies involving human research participants and Sex and Gender in Research.

Reporting on sex and gender	We included sex and age
Population characteristics	See above
Recruitment	Participants with the tumor histologies of interest were included as long as they had available frozen tissue.
Ethics oversight	All tumor samples at DFCI were obtained at the time of resection at Brigham and Women/s Hospital, and were collected under a DFCI/Harvard Cancer Center IRB-approved protocol (01-045) with informed consent of patients. No monetary compensation was offered for patient participation. The samples were then stored fresh frozen in the Gelb Center biobank at the Dana-Farber Cancer Institute, under a protocol approved by the MGB Institutional Review Board.

Note that full information on the approval of the study protocol must also be provided in the manuscript.

Field-specific reporting

Please select the one below that is the best fit for your research. If you are not sure, read the appropriate sections before making your selection.

[x] Life sciences [ ] Behavioural & social sciences [ ] Ecological, evolutionary & environmental sciences

For a reference copy of the document with all sections, see nature.com/documents/nr-reporting-summary-flat.pdf

Life sciences study design

All studies must disclose on these points even when the disclosure is negative.

Sample size	No statistical method is needed to determine sample size for H3K27Ac, H3Kme2, and EPAS1 TF ChIP-seq.<br>All participants with the tumor histologies of interest were included as long as they had available frozen tissue. For epigenetic analyses, 5 or more specimens is sufficient for identifying cistromic changes in human cancers.
Data exclusions	No data exclusion
Replication	Confirmed - FOXA1 Overexpression and EPAS1 knock-down experiments were performed twice independently with reproducible results obtained.
Randomization	This is not relevant to this study as comparisons were performed across distinct clinical states. Samples were allocated into experimental groups based on prior knowledge of the clinical features (histologic subtype of renal cell carcinoma)
Blinding	Immunohistochemistry for BHLHE41, HNF1B, NKX6.1, and ZNF395 was performed in a blinded fashion. All unsupervised analyses described in the study, such as cistrome-wide analyses across states, were blinded as to clinical state of each specimens

Reporting for specific materials, systems and methods

We require information from authors about some types of materials, experimental systems and methods used in many studies. Here, indicate whether each material, system or method listed is relevant to your study. If you are not sure if a list item applies to your research, read the appropriate section before selecting a response.

Materials & experimental systems

n/a	Involved in the study
☐	☒ Antibodies
☐	☒ Eukaryotic cell lines
☒	☐ Palaeontology and archaeology
☒	☐ Animals and other organisms
☐	☒ Clinical data
☒	☐ Dual use research of concern

Methods

n/a	Involved in the study
☐	☒ ChIP-seq
☒	☐ Flow cytometry
☒	☐ MRI-based neuroimaging

Antibodies

Antibodies used	antibodies against mouse HNF1β (Santa Cruz, sc-130407, 1:50 dilution), mouse BHLHE41 (Thermofisher, TA806146, 1:200 dilution), rabbit NKX6.1 (Cell signaling, #54551, 1:50 dilution), rabbit ZNF395 (Lsbio, LS-B5647-100, 1:500 dilution), H3K27ac (Diagenode, C15410196, 1ug/IP dilution), H3K4me2 (Diagenode, C15410035, 1ug/IP dilution), EPAS1 (1:100 dilution, Abcam, ab199)<br><br>Sections were then stained with appropriate secondary antibodies per manufacturer recommendations (ImmPRESS HRP anti-rabbit and anti-mouse IgG Polymer detection kits: MP-7451; MP-7402, Vector laboratories).
Validation	1) HNF1β (Santa Cruz, sc-130407, 1:50 dilution): HNF-1b (94.8) is a mouse monoclonal antibody raised against recombinant HNF-1b of human origin - PMID: 24115587; product datasheet https://datasheets.scbt.com/sc-130407.pdf<br>2) BHLHE41 (Thermofisher, TA806146, 1:200 dilution): SHARP1 (BHLHE41) Mouse Monoclonal Antibody - product datasheet https://cdn.origene.com/datasheet/ta806146.pdf<br>3) NKX6.1 (Cell signaling, #54551, 1:50 dilution): Rabbit mAb recognizes endogenous levels of total NKX6.1 protein - PMID: 35393479; product datasheet https://www.cellsignal.com/products/primary-antibodies/nkx6-1-d8o4r-rabbit-mab/54551<br>4) ZNF395 (Lsbio, LS-B5647-100, 1:500 dilution): Unconjugated rabbit polyclonal antibody to ZNF395 (C-Terminus) from human - product datasheet https://www.lsbio.com/antibodies/ihc-plus-znf395-antibody-c-terminus-ihc-wb-western-ls-b5647/140253<br>5) H3K27Ac (Diagenode, C15410196, 1ug/IP dilution): Polyclonal,ChIP grade, ChIP-seq grade (source: Rabbit) - PMID 30773341; product datasheet - https://www.diagenode.com/files/products/antibodies/Datasheet_H3K27ac_C15410196.pdf<br>6) H3K4me2(Diagenode, C15410035, 1ug/IP dilution): Polyclonal ChIP grade/ChIP-seq grade (source: Rabbit) - PMID: 30244833; product datasheet - https://www.diagenode.com/files/products/antibodies/Datasheet_H3K4me2_pAb-035-050.pdf<br>7) EPAS1 (Abcam, ab199, 1:100 dilution): Rabbit polyclonal to HIF-2-alpha - PMID: 35177645; product datasheet https://www.abcam.com/hif-2-alpha-antibody-ab199.html

Eukaryotic cell lines

Policy information about cell lines and Sex and Gender in Research

Cell line source(s)	786-O cell line was obtained from ATCC
Authentication	786-O cell line was originally purchased from a certified commercial vendor (ATCC). No further authentication was performed.
Mycoplasma contamination	Cell lines tested negative for mycoplasma contamination
Commonly misidentified lines (See ICLAC register)	No commonly misidentified cell lines were used in the study

Clinical data

Policy information about clinical studies

All manuscripts should comply with the ICMJEguidelines for publication of clinical research and a completedCONSORT checklist must be included with all submissions.

Clinical trial registration	Not applicable
Study protocol	Not applicable as this is not a clinical trial
Data collection	Retrospective clinical data was collected and included demographics of the patients such as sex, RCC subtype, grade of tumor and stage of tumor. For the Checkmate 009/010/025, data is publicly available as part of an accompanying paper (Braun et al., Nature Medicine, 2020)
Outcomes	Not applicable

ChIP-seq

Data deposition

☒ Confirm that both raw and final processed data have been deposited in a public database such as GEO.

☒ Confirm that you have deposited or provided access to graph files (e.g. BED files) for the called peaks.

Data access links<br>*May remain private before publication.*	The DFCI datasets generated during and/or analyzed during the current study are available in the GEO repository (GSE188486)
Files in database submission	GSM5683693 K271014T<br>GSM5683694 K271058T<br>GSM5683695 K271091T<br>GSM5683696 K271120T<br>GSM5683697 K271226T<br>GSM5683698 K271247A<br>GSM5683699 K27233T<br>GSM5683700 K27716T<br>GSM5683701 K27951T<br>GSM5683702 K27962T<br>GSM5683703 K27968M<br>GSM5683704 RT0301_H3K27ac<br>GSM5683705 RT0356_H3K27ac<br>GSM5683706 RT0638_H3K27ac<br>GSM5683707 RT0642_H3K27ac<br>GSM5683708 RT1002_H3K27ac<br>GSM5683709 RT1039_H3K27ac<br>GSM5683710 RT1043_H3K27ac<br>GSM5683711 RT1046_H3K27ac<br>GSM5683712 RT1047_H3K27ac<br>GSM5683713 RT1056_H3K27ac<br>GSM5683714 RT1059_H3K27ac<br>GSM5683715 RT1097_H3K27ac<br>GSM5683716 RT1131_H3K27ac<br>GSM5683717 RT1143_H3K27ac<br>GSM5683718 RT1149_H3K27ac<br>GSM5683719 RT1152_H3K27ac<br>GSM5683720 RT1168_H3K27ac<br>GSM5683721 RT1184_H3K27ac<br>GSM5683722 RT1205_H3K27ac<br>GSM5683723 RT1240_H3K27ac<br>GSM5683724 RT1265_H3K27ac<br>GSM5683725 RT929_H3K27ac<br>GSM5683726 RT957_H3K27ac<br>GSM5683727 RT970_H3K27ac<br>GSM5683728 RT991_H3K27ac<br>GSM5683729 RT998_H3K27ac<br>GSM5683730 S20449_H3K27ac<br>GSM5683731 S21381_H3K27ac<br>GSM5683732 S21429_H3K27ac<br>GSM5683733 S22741_H3K27ac<br>GSM5683734 RT0301_H3K4me2<br>GSM5683735 RT0356_H3K4me2<br>GSM5683736 RT0638_H3K4me2<br>GSM5683737 RT0642_H3K4me2<br>GSM5683738 RT1002_H3K4me2<br>GSM5683739 RT1039_H3K4me2<br>GSM5683740 RT1043_H3K4me2<br>GSM5683741 RT1046_H3K4me2<br>GSM5683742 RT1047_H3K4me2<br>GSM5683743 RT1056_H3K4me2<br>GSM5683744 RT1059_H3K4me2<br>GSM5683745 RT1149_H3K4me2<br>GSM5683746 RT1152_H3K4me2<br>GSM5683747 RT1168_H3K4me2<br>GSM5683748 RT1240_H3K4me2<br>GSM5683749 RT1265_H3K4me2

GSM5683750 RT929_H3K4me2
GSM5683751 RT957_H3K4me2
GSM5683752 RT970_H3K4me2
GSM5683753 RT991_H3K4me2
GSM5683754 RT998_H3K4me2
GSM5683755 S20449_H3K4me2
GSM5683756 S21381_H3K4me2
GSM5683757 S22741_H3K4me2
GSM5683758 1039T2AT
GSM5683759 1056T1AT
GSM5683760 1059T1AT
GSM5683761 IN1014T
GSM5683762 IN1058T
GSM5683763 IN1091T
GSM5683764 IN1120T
GSM5683765 IN1226T
GSM5683766 IN1247A
GSM5683767 IN233T
GSM5683768 IN716T
GSM5683769 IN951T
GSM5683770 IN962T
GSM5683771 IN968M
GSM5683772 RT0301_IN
GSM5683773 RT0356_IN
GSM5683774 RT0638_IN
GSM5683775 RT0642_IN
GSM5683776 RT1002_IN
GSM5683777 RT1039_IN
GSM5683778 RT1043_IN
GSM5683779 RT1046_IN
GSM5683780 RT1047_IN
GSM5683781 RT1056_IN
GSM5683782 RT1059_IN
GSM5683783 RT1097_IN
GSM5683784 RT1131_IN
GSM5683785 RT1143_IN
GSM5683786 RT1149_IN
GSM5683787 RT1152_IN
GSM5683788 RT1168_IN
GSM5683789 RT1184_IN
GSM5683790 RT1205_IN
GSM5683791 RT1240_IN
GSM5683792 RT1265_IN
GSM5683793 RT929_IN
GSM5683794 RT957_IN
GSM5683795 RT970_IN
GSM5683796 RT991_IN
GSM5683797 RT998_IN
GSM5683798 S20449_IN
GSM5683799 S21381_IN
GSM5683800 S21429_IN
GSM5683801 S22741_IN
GSM5683802 R0929TAT
GSM5683803 R0957TAT
GSM5683804 R0998TAT
GSM5683805 R1097TAT
GSM5683806 R1149TAT
GSM5683807 R1152TAT
GSM5683808 R991T1AT

Genome browser session (e.g. UCSC)

Not applicable

Methodology

Replicates

Experiments on the 786-O cell line with knock-down and OE were performed in duplicates

Sequencing depth

150bp paired-end sequencing was performed on the Illumina platform. Data shown in Table S26

Antibodies	H3K27ac, Diagenode, C15410196; H3K4me2, Diagenode, C15410035, EPAS1 Abcam ab199
Peak calling parameters	Narrow peaks were called on deduplicated bam files using the following command: macs2 callpeak --SPMR -B -q 0.01 --keep-dup 1 -g hs -f BAMPE --extsize 146 --nomodel -t {treat.bam}.
Data quality	ChIP-seq data were shown to be of high quality by multiple measures, including peak number, fraction of reads in peaks (FRiP score), number of peaks with >10-fold or >20-fold enrichment. narrowPeak calls contained an average of 16047 peaks with >10-fold enrichment. Metrics for individual samples are listed in table S26
Software	MACS v2.1.1.20140616 was used for ChIP-seq peak calling
